# Survival Status of Esophageal Cancer Patients and its Determinants in Ethiopia: A Facility Based Retrospective Cohort Study

**DOI:** 10.3389/fonc.2020.594342

**Published:** 2021-02-15

**Authors:** Hamid Yimam Hassen, Mohammed Ahmed Teka, Adamu Addisse

**Affiliations:** ^1^ Department of Public Health, Faculty of Medicine and Health Sciences, Mizan Tepi University, Mizan Teferi, Ethiopia; ^2^ Department of Primary and Interdisciplinary Care, Faculty of Medicine and Health Sciences, University of Antwerp, Antwerp, Belgium; ^3^ School of Public Health, College of Health Sciences, Addis Ababa University, Addis Ababa, Ethiopia

**Keywords:** survival, esophageal cancer, prognostic determinants, Ethiopia, cohort

## Abstract

**Background:**

Globally, the incidence and mortality due to esophageal cancer are increasing, particularly in low- and middle-income countries. Cancer of the esophagus is the eighth in incidence and seventh in cancer mortality in Ethiopia. A few studies have shown an increasing burden, however, little is known about the survival pattern and its determinants among esophageal cancer patients in Ethiopia. Therefore, we assessed the survival pattern and its determinants among esophageal cancer patients.

**Methods:**

We conducted a retrospective cohort study among 349 esophageal cancer patients who were diagnosed at or referred to Tikur Anbessa Specialized Hospital, Ethiopia from January 2010 to May 2017. Using an abstraction form, nurses who were working at the oncology department extracted the data from patient charts. To estimate and compare the probability of survival among covariate categories, we performed a Kaplan–Meier survival analysis with the log-rank test. To identify the prognostic determinants of survival, we performed a multivariable Cox proportional regression analysis.

**Results:**

The median follow-up time was 32 months with interquartile range of 15 to 42. Overall, the median survival time after diagnosis with esophageal cancer was 4 months with one-, two- and three-year survival of 14.4, 6.3, and 2.4% respectively. In the multivariable Cox proportional hazards model, receiving chemotherapy [Adjusted Hazard Ratio (AHR)=0.36, 95%CI: 0.27–0.49], radiotherapy [AHR=0.38, 95%CI: 0.23–0.63] and surgery [AHR=0.70, 95%CI: 0.54–0.89] were statistically significant.

**Conclusions:**

In Ethiopia, esophageal cancer patients have a very low one-, two- and three-year survival. Despite a very low overall survival, patients who received either chemotherapy, radiotherapy or surgery showed a better survival compared with those who did not receive any treatment. Hence, it is essential to improve the survival of patients with esophageal cancer through early detection and timely initiation of the available treatment options.

## Introduction

Cancer is the second leading cause of morbidity and premature mortality globally, with an estimated 24.5 million new cancer cases and 9.6 million deaths in 2017 (1). Esophageal cancer is ranked ninth in incidence and sixth in cause of cancer deaths in both sexes worldwide, with over a million new cases and 508,585 new deaths in 2018 alone (2).

In the past, non-communicable diseases (NCDs), particularly cancer, were considered as a disease of high-income countries, but recent evidence indicates that it is an important public health issue in low- and middle-income countries (LMICs). A change in lifestyle including sedentary behavior and unhealthy dietary habit, urbanization, cultural transition, and an increase in life expectancy in LMICs might be the possible reason for an increasing incidence (3–5). The largest increase in the incidence of cancer from 2007 to 2017 was observed in middle-income countries (1). By 2030, the cancer burden in sub-Saharan Africa is expected to increase by 85% (6). Similarly, in Ethiopia, the burden of NCDs including cancer is rising. In 2018, with 1,752 estimated new cases, cancer of the esophagus was the eighth most incident and the seventh leading cause of mortality (7, 8). Areas in the African rift valley, particularly Arsi and Bale regions of Ethiopia, Western Kenya, Northern Tanzania and Malawi, are the known hot spots of esophageal cancer (9).

The availability of advanced diagnostic services and early treatment options improve the survival rate in high-income countries. In contrast, in LMICs including Ethiopia the cancer prognosis is very poor, which could be attributable to lack of diagnostic equipment, limited treatment options, and patients visit healthcare at advanced stages (10–13). Hence, the mortality due to cancer, principally cancer of the esophagus is disproportionately higher in LMICs than in high-income countries (14). More than two-third of all cancer deaths happen in LMICs (15).

Esophageal cancer is often associated with an unfavorable prognosis worldwide, with five-year survival ranging from 4 to 40% (14). It is essential to estimate the average survival rate to evaluate and monitor the quality and effectiveness of care provided to cancer patients. However, little is known about the care and management given as well as the survival of patients with cancer in Ethiopia. Although a few studies have been conducted describing the disease burden, little is known on the prognosis of esophageal cancer. Assessment of survival has practical implications for healthcare providers and patients to understand the prognosis over time and for decision making on better treatment options. Thus, this study assessed the overall survival rate and identified its determinants among esophageal cancer patients in Ethiopia.

## Methods and Materials

### Study Setting and Period

This study was conducted at one of the tertiary level hospitals with a cancer diagnostic and treatment facility in Ethiopia named Tikur Anbessa Specialized Hospital (TASH). Under the TASH, the Addis Ababa Population-Based Cancer Registry (AAPBCR) was established in 2011, which serves a catchment population of more than four million inhabitants. The main sources of cases for the registry are pathology centers, hospitals, and higher diagnostic clinics. The time of diagnosis with esophageal cancer was taken as the starting point for follow-up, while the date of death, loss to follow-up, last contact or the end of follow-up time (May 31, 2017) was the end point of the study.

### Study Design and Participants

We conducted a retrospective cohort study among all esophageal cancer patients registered in TASH who were diagnosed or referred from January 1, 2010 to May 31, 2017. The inclusion criteria were all clinically and pathologically confirmed esophageal cancer cases by oncologist. We excluded patient charts with missing information on both histopathology and cancer stage reports. Using the medical record number obtained from the registry, the charts of all esophageal cancer patients were retrieved. Out of 367 charts retrieved, 18 (4.9%) were excluded due to unavailability of neither histopathology nor cancer stage report. Then, we extracted information from 349 patient charts and included them in the analysis.

### Data Collection Procedures

After reviewing literature and consulting experts on important variables, we prepared a data abstraction form considering the availability of information on patient charts and feasibility to get *via* a phone interview. Initially, we identified the charts of all esophageal cancer patients and retrieved using the medical registration number. Then, data collectors reviewed baseline and follow-up patient characteristics including sign and symptoms, laboratory and imaging results, and pathology report.

To ascertain the main outcome, death, the death certificate was identified from the TASH cancer registry. When the death certificate was not available, we did a phone interview with patients or their attendants. Information that was not available from the patient chart or medical register was also collected during the phone interview. In this study, an event was defined as the death of a patient due to esophageal cancer. Patients who were lost to follow-up before developing the event, have incomplete information on the date of death, who died due to other known causes unrelated to esophageal cancer, who do not have registered phone numbers and their current status is unknown, were censored to the last follow-up date. Patients who survived until the last follow-up date were censored to May 31, 2017. Data collection and facilitation of phone interview was conducted by trained oncologic nurses who were working at the oncology center. To improve the data quality, training was given for data collectors on the aim, materials and methods, and data collection procedure for two days.

### Data Processing and Analysis

After checking for completeness, data were coded and entered into EpiInfo version 7.1 and exported to R programming version 3.6.1 for further processing and analysis. For categorical variables, descriptive statistics were computed using frequencies with percentages and rates, whereas continuous variables were summarized using mean with standard deviation (SD) or median with interquartile range (IQR). We calculated the overall death rate from diagnosis to end of follow-up. The variation in overall survival pattern across covariate categories was presented using the Kaplan–Meier curve and tested using the log-rank test. A reverse Kaplan–Meier estimator was used to estimate the median follow-up time (16). We performed a bivariate Cox proportional hazards regression model to identify the crude association of covariates with time to death. Finally, we performed a multivariable Cox regression for ten variables upon checking for the assumptions. Significant multicollinearity was detected between distant metastasis and organ metastasis, then, we excluded the latter from the final model. P-values less than 0.05 in the multivariable Cox proportional hazards model were considered as statistically significant. We presented the results using crude and adjusted Hazard Ratio (HR) with 95% confidence interval (95%CI).

There were 31 (8.9%), five (1.4%), 80 (22.9%), 12 (3.4%), and seven (2.0%) missing values for histology type, tumor location, cancer stage, tobacco use, and family history of cancer respectively. Under missing data at random (MAR) assumption, we managed using a multivariate imputation technique of the ‘**mice**’ package in R (17). We imputed 100 datasets using variables included to the model and additional auxiliary variables. The hazard ratios were estimated in each imputed dataset separately, and combined using Rubin’s rules (18). Missing observations were imputed for the predictor variables used in the multivariable Cox regression model. The outcome variable, death, was not imputed as we analyzed only participants for whom the outcome was ascertained. We performed a sensitivity analysis to assess whether the MAR assumption is valid, and the results were reasonably comparable (**Supplementary Material**).

### Participant Consent and Ethical Approval

The protocol of this study was approved by the institutional review board of Addis Ababa University, College of Health Sciences. Before starting the phone interview, informed consent was obtained from patients or caretakers. This study is in compliance with the principles of the declaration of Helsinki. The confidentiality of patients’ data was kept at each step of data collection and processing.

## Results

### Sociodemographic and Behavioral Characteristics of Patients

The sociodemographic and behavioral characteristics of patients are summarized in **Table 1**. The mean age of patients was 51.4 years (SD: 11.9), and 206 (59.0%) were females. More than half (56.7%) were from the Oromia region and 319 (91.4%) were married. Sixty-two (17.8%), 18 (5.3%), and 92 (26.4%) had a history of alcohol intake, smoking and Khat (*Catha edulis*) chewing respectively. The prevalence of alcohol consumption significantly varied across gender, in which 9.2% of females and 30.1% of males had a history of alcohol intake (p < 0.001). Similarly, none of female participants reported tobacco use, whereas 15.4% of males use tobacco and the difference was statistically significant (p < 0.001). Moreover, the prevalence of Khat chewing was significantly higher among males (35.0%) than females (20.4%) (p = 0.004).

**Table 1:** Sociodemographic and behavioral characteristics of patients with esophageal cancer in TASH, Addis Ababa, Ethiopia, 2010–2017 (n = 349).

Variables	Frequency	Percent
**Age** (years) (mean/SD)	51.4	11.9
**Sex**		
Male	143	41.0
Female	206	59.0
**Marital status**		
Single	10	2.9
Married	319	91.4
Widowed	14	4.0
Divorced	6	1.7
**Residence region**		
Addis Ababa	54	15.5
Amhara	26	7.4
Oromia	198	56.7
SNNPR	54	15.5
Others^¥^	17	4.9
**Consume alcohol**	62	17.8
**Chew Khat**	92	26.4
**Tobacco use** (n = 337)	18	5.3
**Has family history of cancer** (n = 342)	2	0.6

^¥^Dire Dawa, Harari, Gambella, Somali, Afar, Tigray.

TASH, Tikur Anbesa Specialized Hospital.

### Histologic Types, Anatomic Site, and Stage of Esophageal Cancer

Out of 349 cases registered, 318 (91.1%) and 57 (16.3%) of patient charts had histological type of cancer and histologic grade report, respectively. Among those with histopathology test results, 287 (90.3%) were squamous cell carcinoma, whereas 31 (9.7%) were adenocarcinoma type. Over half (54.1%) of the cases had lesions at the lower third of the esophagus, whereas 105 (30.5%) at the middle third. Two hundred sixty nine (77.1%) of charts had reports of cancer stage at diagnosis, of which 188 (69.9%) and 51 (19.0%), respectively, were stages IV and III at diagnosis (**Table 2**).

**Table 2:** Distribution of histologic types and histologic grades of patients with esophageal cancer in TASH, Addis Ababa, Ethiopia, 2010–2017 (n = 349).

Variables	Frequency	Percent
**Histological type** (n = 318)		
Squamous-cell carcinoma	287	90.3
Adenocarcinoma	31	9.7
**Tumor location** (n = 344)		
Upper third	53	15.4
Middle third	105	30.5
Lower third	186	54.1
**Histological grade** (n = 57)		
Well differentiated	38	66.7
Moderately differentiated	10	17.5
Poorly differentiated	6	10.5
Undifferentiated	3	5.3
**Stage at diagnosis** (n = 269)		
Stage I	3	1.1
Stage II	27	10.0
Stage III	51	19.0
Stage IV	188	69.9

TASH, Tikur Anbesa Specialized Hospital.

### Treatment Options Given to Patients

Out of 349 patients, 183 (52.1%) were treated with trans-hiatal esophagectomy surgical procedure, while 112 (31.9%) transthoracic esophagectomy, and 112 (31.9%) were managed using feeding gastrostomy. Above one-fourth (25.8%) of them received chemotherapy, whereas only 26 (7.7%) were treated with radiotherapy (**Table 3**).

**Table 3:** Treatment options given to esophageal cancer patients at TASH, Addis Ababa, Ethiopia, 2010–2017 (n = 349).

Treatment received	Frequency	Percent
**Surgery**	183	52.4
**Type of surgery** (n = 183)		
Trans-hiatal esophagectomy	145	79.2
Trans-thoracic esophagectomy	29	15.8
Feeding gastrostomy	112	61.2
Laparotomy	71	38.8
**Chemotherapy**	89	25.5
Type of chemotherapy (n = 90)		
Neoadjuvant	3	3.4
Adjuvant	70	78.6
Palliative	17	19.1
**Radiotherapy**	26	7.4
**Type of radiotherapy** (n = 26)		
Adjuvant	2	7.7
Radical	1	3.8
Palliative	23	88.5

TASH, Tikur Anbesa Specialized Hospital.

### Survival Time From Diagnosis to Death

**Figure 1:**
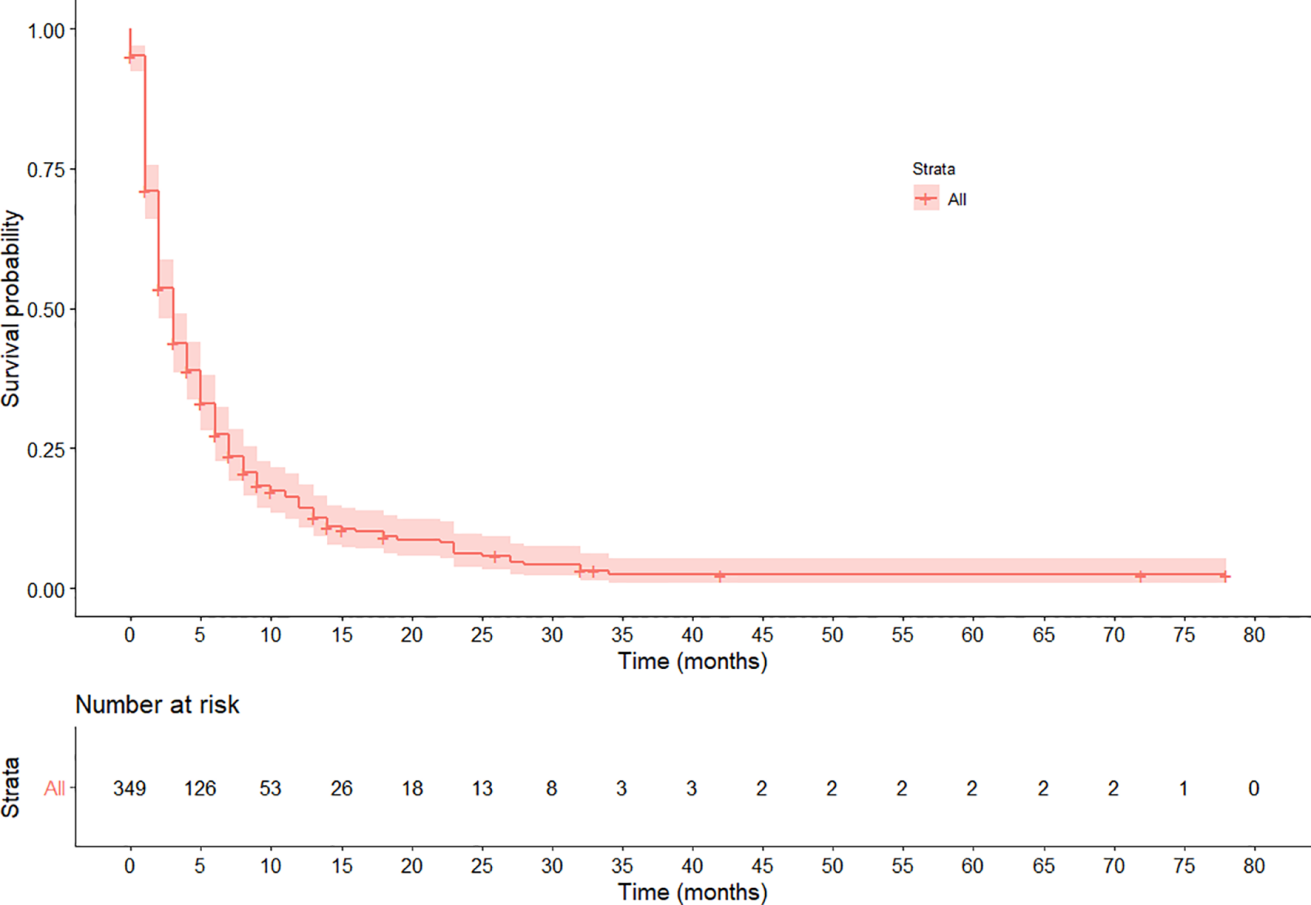
Kaplan–Meier survival curve of the overall survival pattern among esophageal cancer patients registered in Tikur Anbessa Specialized Hospital, Addis Ababa, Ethiopia, 2010–2017. The curve shows the median survival is 4 months.

The median follow-up time was 32 months with IQR of 15 to 42 months. Three-hundred ten (88.8%) patients died during the 1,932 person-month follow-up period, resulting in an overall event rate of 160.5 per 1,000 person**–**months [95%CI: 119.2–320.6]. The overall survival rate was very low with one-, two- and three-year survival rates of 14.4% [95%CI: 11.0–18.9], 6.3% [3.9%–10.2], and 2.4% [0.9%–6.0%] respectively, and the median survival time was 4 months [95%CI: 2–8] (**Figure 1**).

### Variation in Survival Rates Among Groups of Esophageal Cancer Patients

**Figure 2:**
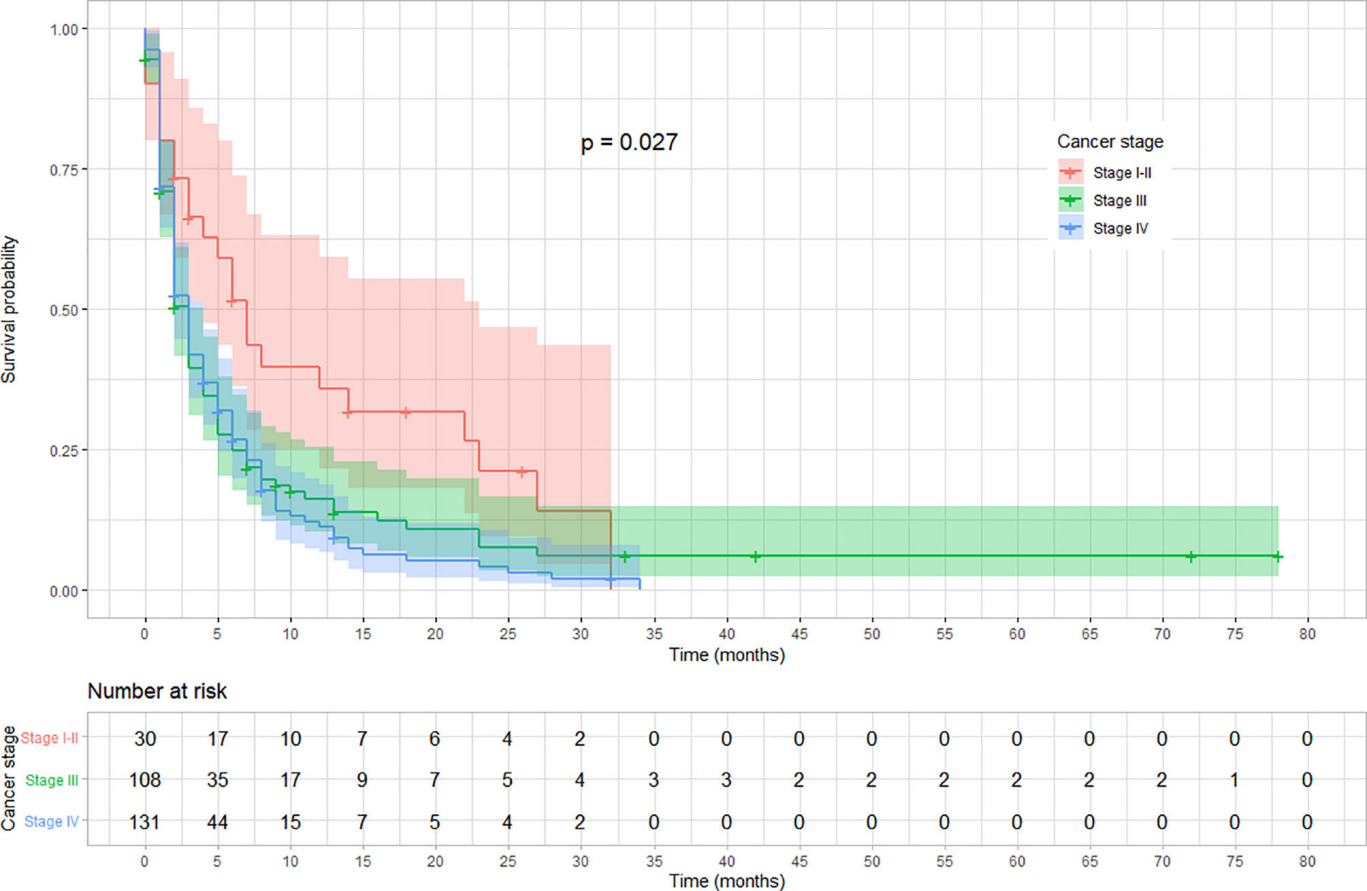
Kaplan–Meier survival curve showing the variation in overall survival based on cancer stage at diagnosis among esophageal cancer patients in Ethiopia, 2010–2017 (Log-rank test, *p* = 0.027).

**Figure 3:**
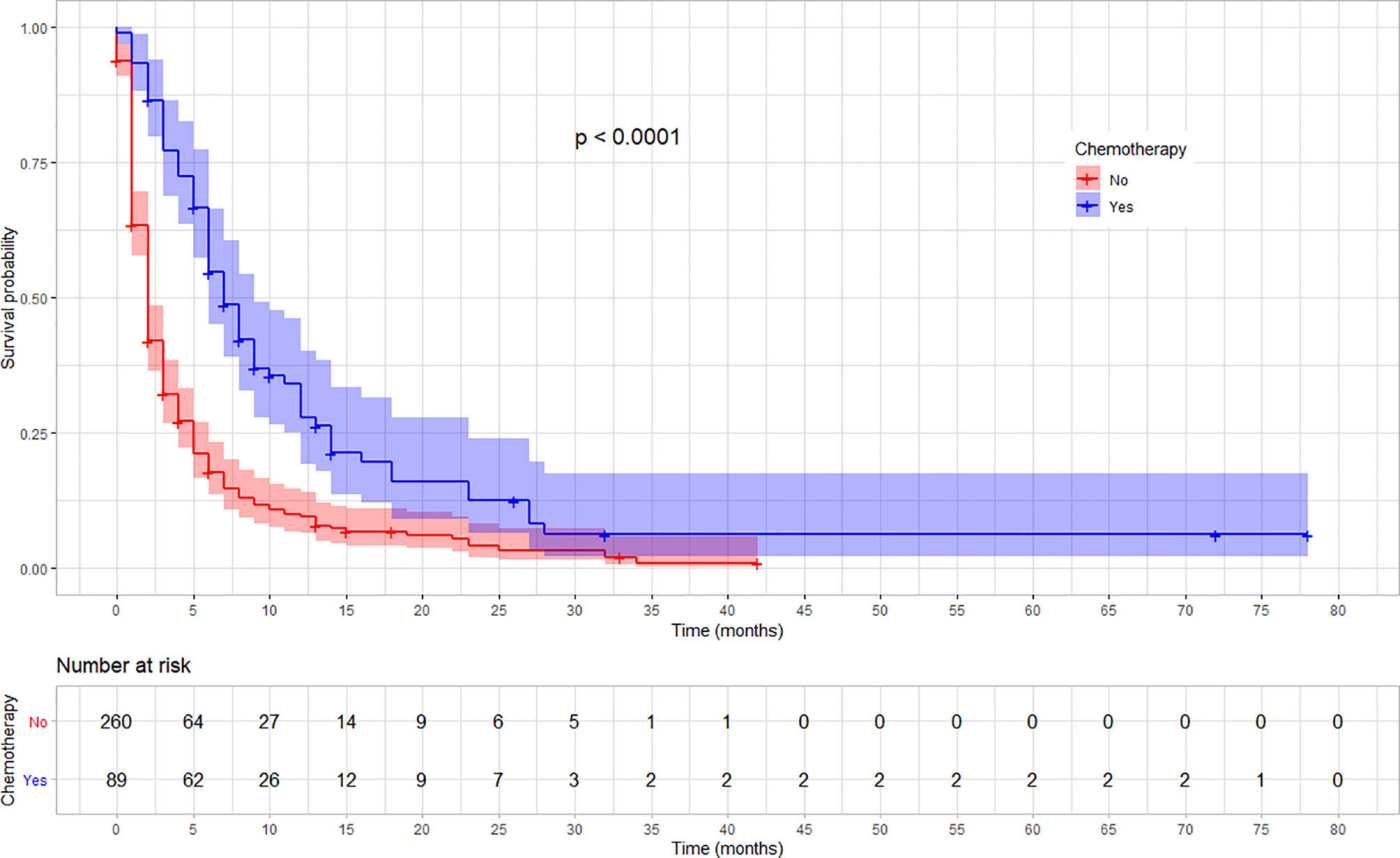
Kaplan–Meier survival curve showing the difference in overall survival based on the chemotherapy treatment among esophageal cancer patients in Ethiopia, 2010–2017 (Log-rank, *p* < 0.01).

**Figure 4:**
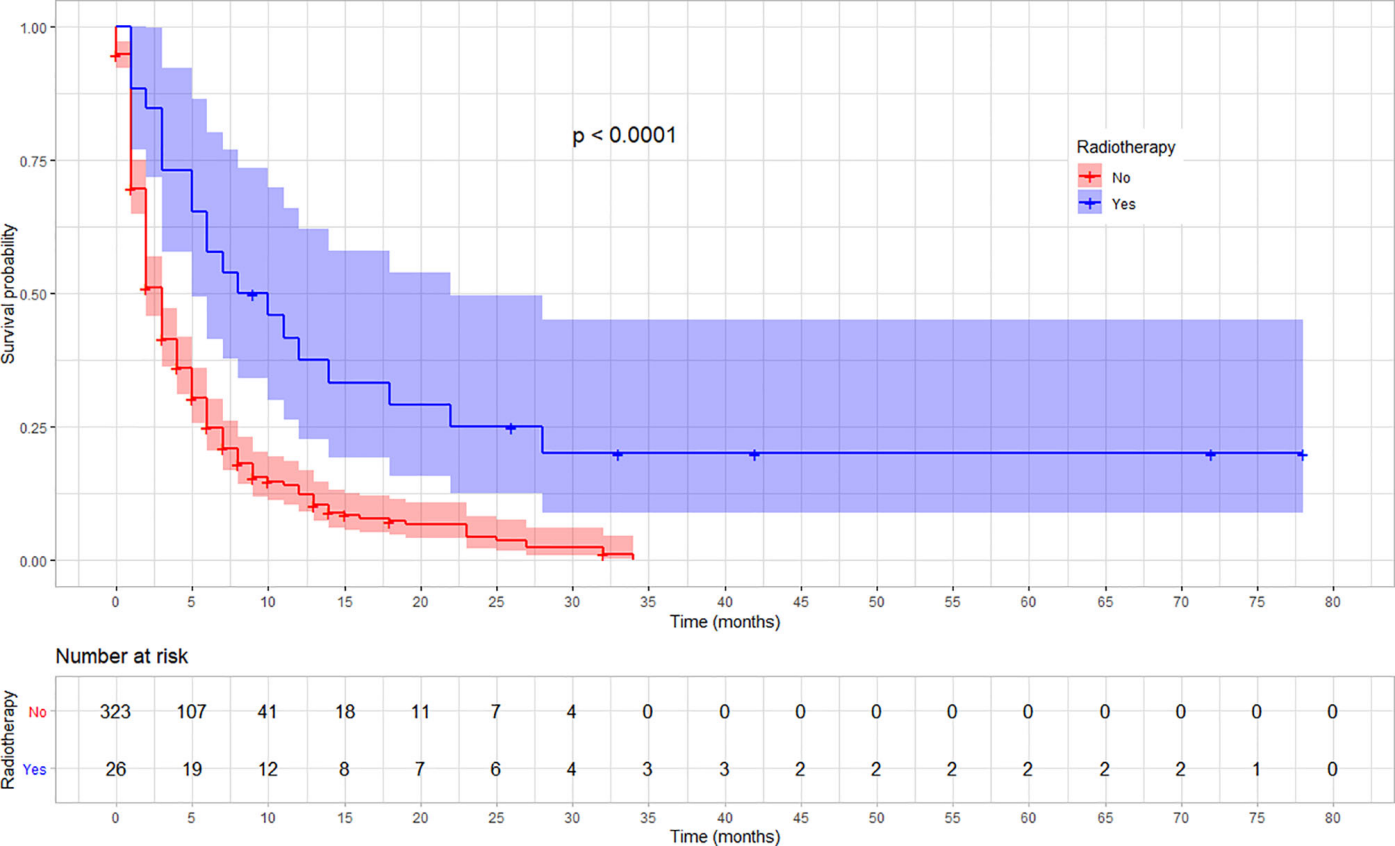
Kaplan–Meier survival curve showing the difference in overall survival by radiotherapy treatment status among esophageal cancer patients in Ethiopia, 2010–2017. (Log-rank test, *p* < 0.001).

The rate of survival varied across categories of covariates such as the stage of cancer, chemotherapy, and radiotherapy treatment status. The survival varied along with the cancer stage, with lower stages at diagnosis showing a better survival (log-rank test, p < 0.03). Similarly, patients who received chemotherapy showed a better overall survival compared with those who did not (log-rank test p < 0.001). Moreover, the overall survival was significantly different among patients based on their radiotherapy treatment status, in which those who received showed a better survival (log-rank test, p < 0.001). No significant variation was observed on overall survival according to sex and location of the tumor (p = 0.057). The variation in survival pattern among covariate categories is presented in **Figures 2**–**4**.

### Prognostic Determinants of Survival Among Esophageal Cancer Patients

In the multivariable Cox proportional hazards model, receiving chemotherapy, radiotherapy, and surgery independently determine the survival from esophageal cancer. The death rate decreased by 64% for those patients who received chemotherapy compared with those who did not (AHR = 0.36, 95%CI: 0.27–0.49). Similarly, those who were treated with radiotherapy had 62% lower rate of death than those who did not (AHR = 0.38, 95%CI: 0.23–0.63). The death rate was also 30% lower for patients who were treated with any type of surgery in comparison with those who did not (AHR = 0.70, 95%CI: 0.54–0.89). However, there was no statistically significant interaction between any of the predictors. Age, baseline hemoglobin, sex, and histology type were not statistically significant in the multivariable Cox regression (**Table 4**).

**Table 4:** Cox proportional hazards model of the determinants of survival among esophageal cancer patients registered at TASH, Addis Ababa, Ethiopia, 2010–2017.

**Determinants**	**CHR (95%CI)**	**AHR (95%CI)**
**Age**	1.01 (0.99–1.02)	1.01 (0.99–1.02)
**Sex** *(female)*	1.17 (0.93–1.48)	1.06 (0.84–1.36)
**Distant metastasis** *(yes)*	1.30 (0.98–1.71)	1.16 (0.85–1.59)
**Histology type**		
Squamous-cell carcinoma	1	1
Adenocarcinoma	0.93 (0.64–1.36)	0.79 (0.52–1.18)
**Cancer stage**		
I and II	1	1
III	1.37 (0.92–2.02)	1.16 (0.76–1.75)
IV	1.48 (1.02–2.15)*	1.28 (0.84–1.94)
**Tumor location**		
Upper	1	1
Middle	1.40 (0.99–1.99)	0.99 (0.68–1.44)
Lower	1.07 (0.78–1.48)	0.74 (0.52–1.07)
**Chemotherapy** *(yes)*	0.43 (0.33–0.56)**	0.36 (0.27–0.49)**
**Baseline hemoglobin**	1.03 (0.98–1.09)	1.02 (0.97–1.08)
**Surgery** *(yes)*	0.87 (0.69–1.09)	0.70 (0.54–0.89)*
**Radiotherapy** *(yes)*	0.39 (0.24–0.62)**	0.38 (0.23–0.63)**

*P < 0.05; ** P < 0.01.

*AHR, Adjusted Hazard Ratio; CHR, Crude Hazard Ratio; CI, Confidence Interval;* TASH, Tikur Anbesa Specialized Hospital.

Multivariate multiple imputations were performed (n = 349).

## Discussion

In this study, we highlighted the survival pattern and prognostic determinants of esophageal cancer among patients who were diagnosed at or referred to Tikur Anbessa Specialized Hospital. Such a study has not been reported from Ethiopia to date. We found that the overall survival after diagnosed with esophageal cancer was very low. Despite very low overall survival, those who received either chemotherapy, radiotherapy, or surgery showed a better survival compared with those who did not.

In Ethiopia, the survival from all types of cancer is relatively low in comparison with high-income countries. A study by Beksisa and his colleagues showed a three-year survival of prostate cancer was estimated to be 38.9% (19). Similarly, a study from other parts of the country showed a two-year survival of breast cancer to be 53% (20). Several studies in other parts of the world indicated esophageal cancer has a poor prognosis in comparison with other types of cancers (21, 22). Hence, the survival from esophageal cancer is expected to be worse in LMICs including Ethiopia.

In our study, the overall one-, two- and three-year survival for all stages combined was below 15%. This finding is lower than the rate reported by a study done in Brazil, which showed a 22.8 and 20.2% five-year survival for squamous and adenocarcinoma, respectively (23). Moreover, in our study the median survival time after diagnosis was 4 months, which is in line with a study from Mozambique which reported 3.5 months (24). On the other hand, a study in Cameroon and Tanzania reported a relatively higher median survival of 6.7 and 6.9 months respectively (25, 26). In our study, patients visited healthcare at later stages of the disease, majorities (89%) were diagnosed either at stage III or IV. The lower survival could also be attributed to the lower socio-development index (SDI) of the country (27). A study by Wong and his associates indicated the incidence and mortality of esophageal cancer is highly correlated with SDI of countries (28). The economic development of a country determines the patient’s health seeking behavior and lifestyle, access to screening and management options, which in turn impact the survival from esophageal cancer (29, 30).

In our study, patients who received chemotherapy have a 64% lower probability of death, supporting the hypothesis that chemotherapy is an efficacious treatment option for advanced esophageal cancer. Coherently, a study in China showed esophagectomy and chemo-radiotherapy were associated with a better survival (31). A systematic review in Africa also reported consistent results (32).

Furthermore, the rate of death is 62% lower among patients who were treated using radiotherapy than those who did not. Similar studies indicated radiotherapy improves survival from esophageal cancer (33, 34). Hence, expansion of radiotherapy centers and training of skilled professionals could help to reduce mortality from esophageal as well as other types of cancer.

This study also showed that patients treated with surgery had a 30% lower rate of death than their counterparts, which is supported by a systematic review that showed the best treatment options to be esophagectomy with a 3-year survival rate of 76.6% (32). Consistently, a study in Kenya indicated patients treated with esophagectomy had a better survival compared to intubations (35). A study in Japan also showed the 5-year survival rates for patients who undertook surgery and those who did not were 17 and 13%, respectively, indicating the importance of surgery (36).

As part of the strength of this study, we used a multivariable Cox regression, which allowed estimating survival patterns of patients with an unequal follow-up period and also took account of censoring. Furthermore, due to the inclusion of all the patients who fulfilled the eligibility criteria, sampling error was avoided or minimized. In addition, in our study more than three hundred patients experienced the event, which made our Kaplan**–**Meier and Cox regression estimates more precise. Harrel et al. (37) suggest that the Cox regression model needs a minimum of 10 events per each covariate in the model, indicating our analysis is sufficiently powered to identify determinants of survival. However, the following limitations need to be considered in interpretation of findings. First, since we used existing patient charts, data were missed for some variables, particularly histological grade. Nevertheless, we managed missing data using multiple imputation, which provided more precise estimates. Second, confirmation of death and its cause for some of the patients used verbal autopsy through phone interview, which may not be as accurate as hospital death records or vital event registrations. As a result deaths due to esophageal cancer might be overestimated, leading to outcome ascertainment bias. Nevertheless, since the misclassification is independent of the prognostic factors, the effect on the hazard ratios is negligible. At last, since the majority of patients were diagnosed at an advanced stage of cancer, the overall median survival was too small, leading to the estimation of time specific survival rates being less precise. Further determinants of survival could be identified using studies which recruit larger sample size.

## Conclusions

This study identified, in Ethiopia, patients diagnosed with esophageal cancer have a very low survival rate. The death rate due to esophageal cancer is significantly different according to the stage of cancer at diagnosis and treatment modalities they received, radiotherapy, chemotherapy, and/or surgery. Patients diagnosed at an advanced cancer stage and those who did not receive either of the treatment options showed lower survival rate. These indicate early diagnosis and timely initiation of the available treatment options are essential to improve survival of patients with esophageal cancer. Hence, improvements in cancer control programs, including screening, prevention, timely initiation of available treatment, and establishment of comprehensive cancer registry are recommended. Moreover, public health experts should collaborate with clinicians and community leaders to increase awareness on prevention strategies and early symptoms of esophageal cancer to assist early visit to healthcare. To improve utility of data for further research and policy, healthcare providers working at oncology units need to give more attention to document all relevant patient information on the medical record and the cancer registry. We recommend future studies employing prospective design and larger samples.

## Data Availability Statement

The data that support the findings of this study are available upon request from the authors.

## Ethics Statement

The studies involving human participants were reviewed and approved by the institutional review board of Addis Ababa University, College of Health Sciences. The patients/participants provided their written informed consent to participate in this study.

## Author Contributions

HH: Conceptualization, methodology, formal analysis, software, validation, investigation, data curation, visualization, supervision, writing—original draft, writing—reviewing and editing. MT: Conceptualization, methodology, validation, investigation, resources, data curation, visualization, supervision, project administration, funding acquisition, writing—reviewing and editing. AA: Methodology, supervision, project administration, validation, resources, writing—reviewing and editing. All authors contributed to the article and approved the submitted version.

## Funding

MT got partial financial support from Addis Ababa University. All funders had no role in the study design, data collection and analysis, decision to publish, or preparation of the manuscript. There was no additional external funding received for this study.

## Conflict of Interest

The authors declare that the research was conducted in the absence of any commercial or financial relationships that could be construed as a potential conflict of interest.

## References

[1] Fitzmaurice C Allen C Barber RM Barregard L Bhutta ZA Brenner H. Global, Regional, and National Cancer Incidence, Mortality, Years of Life Lost, Years Lived With Disability, and Disability-Adjusted Life-years for 32 Cancer Groups, 1990 to 2015: A Systematic Analysis for the Global Burden of Disease Study. JAMA Oncol (2017) 3(4):524–48. 10.1001/jamaoncol.2016.5688 PMC6103527 27918777

[2] Bray F Ferlay J Soerjomataram I Siegel RL Torre LA Jemal A. Global cancer statistics 2018: GLOBOCAN estimates of incidence and mortality worldwide for 36 cancers in 185 countries. CA: A Cancer J Clin (2018) 68(6):394–424. 10.3322/caac.21492 30207593

[3] Freddie Bray Isabelle S. “The Changing Global Burden of Cancer: Transitions in Human Development and Implications for Cancer Prevention and Control”. In: Disease Control Priorities, 3rd ed. The World Bank (2015). vol. 3. p. 23–44. Cancer. 26913347

[4] Mallath MK Taylor DG Badwe RA Rath GK Shanta V Pramesh CS. The growing burden of cancer in India: epidemiology and social context. Lancet Oncol (2014) 15(6):e205–e12. 10.1016/S1470-2045(14)70115-9 24731885

[5] McCormack VA Boffetta P. Today’s lifestyles, tomorrow’s cancers: trends in lifestyle risk factors for cancer in low- and middle-income countries. Ann Oncol (2011) 22(11):2349–57. 10.1093/annonc/mdq763 21378201

[6] American Cancer Society. The history of cancer. Available at: https://www.cancer.org/cancer/cancer-basics/history-of-cancer.html, May 12, 2019.

[7] International Agency for Research on Cancer, Organization WH. Globocan. Cancer fact sheets, Ethiopia 2018. Globocan (2018). Available at: https://gco.iarc.fr/today/data/factsheets/populations/231-ethiopia-fact-sheets.pdf.

[8] Memirie ST Habtemariam MK Asefa M Deressa BT Abayneh G Tsegaye B. Estimates of Cancer Incidence in Ethiopia in 2015 Using Population-Based Registry Data. J Global Oncol (2018) 4:1–11. 10.1200/JGO.17.00175 PMC6223441 30241262

[9] Schaafsma T Wakefield J Hanisch R Bray F Schüz J Joy EJM. Africa’s Oesophageal Cancer Corridor: Geographic Variations in Incidence Correlate with Certain Micronutrient Deficiencies. PLoS One (2015) 10(10):e0140107–e. 10.1371/journal.pone.0140107 PMC4598094 26448405

[10] Ferlay J Soerjomataram I Dikshit R Eser S Mathers C Rebelo M. Cancer incidence and mortality worldwide: sources, methods and major patterns in GLOBOCAN 2012. Int J Cancer (2015) 136(5):E359–86. 10.1002/ijc.29210 25220842

[11] Torre LA Bray F Siegel RL Ferlay J Lortet-Tieulent J Jemal A. Global cancer statistics, 2012. CA Cancer J Clin (2015) 65(2):87–108. 10.3322/caac.21262 25651787

[12] Jemal A Bray F Forman D O’Brien M Ferlay J Center M. Cancer burden in Africa and opportunities for prevention. Cancer (2012) 118(18):4372–84. 10.1002/cncr.27410 22252462

[13] Fadlelmola FM. Cancer registries and cancer genomics research in east Africa: challenges and lessons learned. Int Clin Pathol J (2016) 2(4):67–76. 10.15406/icpjl.2016.02.00045

[14] Fitzmaurice C Dicker D Pain A Hamavid H Moradi-Lakeh M MacIntyre MF. The Global Burden of Cancer 2013. JAMA Oncol (2015) 1(4):505–27. 10.1001/jamaoncol.2015.0735 PMC4500822 26181261

[15] World Health Organization. WHO. Cancer fact sheet. (2018). Available at: https://www.who.int/news-room/fact-sheets/detail/cancer.

[16] Kaplan EL Meier P. Nonparametric Estimation from Incomplete Observations. J Am Stat Assoc. (1958) 53(282):457–81. 10.2307/2281868

[17] van Buuren S Groothuis-Oudshoorn K. mice: Multivariate imputation by chained equations in R. J Stat Softw (2011) 1(3):1–68. 10.18637/jss.v045.i03

[18] Rubin DB. Multiple imputation for nonresponse in surveys. New Jersey, United States: John Wiley & Sons (2004).

[19] Beksisa J Getinet T Tanie S Diribi J Hassen HY. Survival and prognostic determinants of prostate cancer patients in Tikur Anbessa Specialized Hospital, Addis Ababa, Ethiopia: A retrospective cohort study. PLoS One (2020) 15(3):e0229854. 10.1371/journal.pone.0229854 32134996 PMC7058322

[20] Eber-Schulz P Tariku W Reibold C Addissie A Wickenhauser C Fathke C. Survival of breast cancer patients in rural Ethiopia. Breast Cancer Res Treat (2018) 170(1):111–8. 10.1007/s10549-018-4724-z 29479644

[21] American Cancer Society. Cancer Facts and Figures 2019. Atlanta: American Cancer Society (2019).

[22] Berrino F De Angelis R Sant M Rosso S Lasota MB Coebergh JW. Survival for eight major cancers and all cancers combined for European adults diagnosed in 1995–99: results of the EUROCARE-4 study. Lancet Oncol (2007) 8(9):773–83. 10.1016/S1470-2045(07)70245-0 17714991

[23] Tustumi F Kimura CMS Takeda FR Uema RH Salum RAA Ribeiro-Junior U. Prognostic factors and survival analysis in esophageal carcinoma. Arq Bras Cir Dig (2016) 29(3):138–41. 10.1590/0102-6720201600030003 PMC5074661 27759773

[24] Come J Castro C Morais A Cossa M Modcoicar P Tulsidâs S. Clinical and Pathologic Profiles of Esophageal Cancer in Mozambique: A Study of Consecutive Patients Admitted to Maputo Central Hospital. J Glob Oncol (2018) 4:1–9. 10.1200/jgo.18.00147 PMC7010456 30398947

[25] Nga WTB Eloumou S Engbang JPN Bell EMD Mayeh AMM Atenguena E. Prognosis and survival of esophageal cancer in Cameroon: a prognostic study. Pan Afr Med J (2019) 33:73. 10.11604/pamj.2019.33.73.16112 31448035 PMC6689847

[26] Mmbaga EJ Deardorff KV Mushi B Mgisha W Merritt M Hiatt RA. Characteristics of Esophageal Cancer Cases in Tanzania. J Glob Oncol (2018) 4:1–10. 10.1200/jgo.2016.006619 PMC6180793 30241222

[27] Pakzad R Mohammadian-Hafshejani A Khosravi B Soltani S Pakzad I Mohammadian M. The incidence and mortality of esophageal cancer and their relationship to development in Asia. Ann Transl Med (2016) 4(2):29–. 10.3978/j.issn.2305-5839.2016.01.11 PMC4731602 26889482

[28] Wong MCS Hamilton W Whiteman DC Jiang JY Qiao Y Fung FDH. Global Incidence and mortality of oesophageal cancer and their correlation with socioeconomic indicators temporal patterns and trends in 41 countries. Sci Rep (2018) 8(1):4522. 10.1038/s41598-018-19819-8 29540708 PMC5852053

[29] Merletti F Galassi C Spadea T. The socioeconomic determinants of cancer. Environ Health (2011) 10(1):S7. 10.1186/1476-069X-10-S1-S7 21489217 PMC3073199

[30] Naik H Qiu X Brown MC Eng L Pringle D Mahler M. Socioeconomic status and lifestyle behaviours in cancer survivors: smoking and physical activity. Curr Oncol (2016) 23(6):e546–e55. 10.3747/co.23.3166 PMC5176380 28050143

[31] Cai W Ge W Yuan Y Ding K Tan Y Wu D. A 10-year Population-based Study of the Differences between NECs and Carcinomas of the Esophagus in Terms of Clinicopathology and Survival. J Cancer (2019) 10(6):1520–7. 10.7150/jca.29483 PMC6485230 31031862

[32] Asombang AW Chishinga N Nkhoma A Chipaila J Nsokolo B Manda-Mapalo M. Systematic review and meta-analysis of esophageal cancer in Africa: Epidemiology, risk factors, management and outcomes. World J Gastroenterol (2019) 25(31):4512–33. 10.3748/wjg.v25.i31.4512 PMC6710188 31496629

[33] Zhang W Luo Y Wang X Han G Wang P Yuan W. Dose-escalated radiotherapy improved survival for esophageal cancer patients with a clinical complete response after standard-dose radiotherapy with concurrent chemotherapy. Cancer Manag Res (2018) 10:2675–82. 10.2147/CMAR.S160909 PMC6097517 30147366

[34] Di Fiore F Lecleire S Rigal O Galais MP Ben Soussan E David I. Predictive factors of survival in patients treated with definitive chemoradiotherapy for squamous cell esophageal carcinoma. World J Gastroenterol (2006) 12(26):4185–90. 10.3748/wjg.v12.i26.4185 PMC4087370 16830371

[35] Ogendo SW. Follow up of oesophageal cancer therapy at the Kenyatta National Hospital, Nairobi. East Afr Med J (2001) 78(12):650–4. 10.4314/eamj.v78i12.8935 12199447

[36] Fujita H Sueyoshi S Tanaka T Tanaka Y Sasahara H Shirouzu K. Prospective non-randomized trial comparing esophagectomy-followed-by-chemoradiotherapy versus chemoradiotherapy-followed-by-esophagectomy for T4 esophageal cancers. J Surg Oncol (2005) 90(4):209–19. 10.1002/jso.20259 15906363

[37] Harrell FE Jr. Regression modeling strategies: with applications to linear models, logistic and ordinal regression, and survival analysis. Berlin, Germany: Springer (2015).

