# Supplementary material for: Survival Status of Esophageal Cancer Patients and its Determinants in Ethiopia: A Facility Based Retrospective Cohort Study
Source: Front Oncol. 2021 Feb 15;10:594342. doi: 10.3389/fonc.2020.594342 (PMC7917207; doi:10.3389/fonc.2020.594342)
Supplement: Supplementary file 1 [file Table_1.docx]

**Annex 1. Sensitivity analysis: comparison of the Cox proportional hazards model in the imputed dataset using multiple imputation and complete case analysis on determinants of survival among patients with esophageal cancer at Tikur Anbessa Specialized Hospital, Addis Ababa, 2012–2016**

| **Determinants** | **Complete case analysis**  **AOR (95%CI)** | **Multiple imputation**  **AHR (95%CI)** |
| --- | --- | --- |
| ***Age*** | 1.01 (0.99–1.03) | 1.01 (0.99–1.02) |
| ***Sex*** | | |
| Male | 1 | 1 |
| Female | 0.97 (0.66–1.42) | 1.06 (0.84–1.36) |
| ***Distant metastasis*** | | |
| No | 1 | 1 |
| Yes | 1.43 (0.84–2.44) | 1.16 (0.85–1.59) |
| ***Histology type*** | | |
| SCC | 1 | 1 |
| AC | 0.74 (0.41–1.36) | 0.79 (0.52–1.18) |
| ***Cancer stage*** | | |
| I and II | 1 | 1 |
| III | 1.71 (0.58–3.61) | 1.16 (0.76–1.75) |
| IV | 2.10 (0.95–4.66) | 1.28 (0.84–1.94) |
| ***Tumor location*** | | |
| Upper | 1 | 1 |
| Middle | 1.07 (0.59–1.96) | 0.99 (0.68–1.44) |
| Lower | 0.87 (0.47–1.61) | 0.74 (0.52–1.07) |
| ***Chemotherapy*** | | |
| No | 1 | 1 |
| Yes | 0.40 (0.27–0.61)***** | 0.36 (0.27–0.49)** |
| ***Baseline Hemoglobin*** | 1.04 (0.96–1.13) | 1.02 (0.97–1.08) |
| ***Surgery*** | | |
| No | 1 | 1 |
| Yes | 0.81 (0.55–1.20) | 0.70 (0.54–0.89)* |
| ***Radiotherapy*** | | |
| No | 1 | 1 |
| Yes | 0.24 (0.10–0.58)****** | 0.38 (0.23–0.63)** |

**P<0.05; ** P<0.01*

*CHR: Crude Hazard Ratio; AHR: Adjusted Hazard Ratio*
